# Divisions of labor in the thiamin biosynthetic pathway among organs of maize

**DOI:** 10.3389/fpls.2014.00370

**Published:** 2014-08-04

**Authors:** Jiahn-Chou Guan, Ghulam Hasnain, Timothy J. Garrett, Christine D. Chase, Jesse Gregory, Andrew D. Hanson, Donald R. McCarty

**Affiliations:** ^1^Genetics Institute and Horticultural Sciences Department, Institute of Food and Agricultural Sciences, University of Florida Gainesville, FL, USA; ^2^Horticultural Sciences Department, Institute of Food and Agricultural Sciences, University of Florida Gainesville, FL, USA; ^3^Department of Pathology, Immunology, and Laboratory Medicine, College of Medicine, University of Florida Gainesville, FL, USA; ^4^Department of Food Science and Human Nutrition, Institute of Food and Agricultural Sciences, University of Florida Gainesville, FL, USA

**Keywords:** thiamin biosynthesis, comparative transcriptomics, maize development, pollen development, meristem metabolism

## Abstract

The B vitamin thiamin is essential for central metabolism in all cellular organisms including plants. While plants synthesize thiamin *de novo*, organs vary widely in their capacities for thiamin synthesis. We use a transcriptomics approach to appraise the distribution of *de novo* synthesis and thiamin salvage pathways among organs of maize. We identify at least six developmental contexts in which metabolically active, non-photosynthetic organs exhibit low expression of one or both branches of the *de novo* thiamin biosynthetic pathway indicating a dependence on inter-cellular transport of thiamin and/or thiamin precursors. Neither the thiazole (THI4) nor pyrimidine (THIC) branches of the pathway are expressed in developing pollen implying a dependence on import of thiamin from surrounding floral and inflorescence organs. Consistent with that hypothesis, organs of the male inflorescence and flowers are shown to have high relative expression of the thiamin biosynthetic pathway and comparatively high thiamin contents. By contrast, divergent patterns of THIC and THI4 expression occur in the shoot apical meristem, embyro sac, embryo, endosperm, and root-tips suggesting that these sink organs acquire significant amounts of thiamin via salvage pathways. In the root and shoot meristems, expression of THIC in the absence of THI4 indicates a capacity for thiamin synthesis via salvage of thiazole, whereas the opposite pattern obtains in embryo and endosperm implying that seed storage organs are poised for pyrimidine salvage. Finally, stable isotope labeling experiments set an upper limit on the rate of *de novo* thiamin biosynthesis in maize leaf explants. Overall, the observed patterns of thiamin biosynthetic gene expression mirror the strategies for thiamin acquisition that have evolved in bacteria.

## Introduction

Thiamin (vitamin B1), in its diphosphate form, is an essential co-factor for key enzymes of central metabolism in cellular organisms. Among the eukaryotes, plants and fungi have a capacity for *de novo* synthesis of thiamin as well as other B vitamins (Gerdes et al., 2012). The thiamin biosynthetic pathway is comprised of separate branches for synthesis of pyrimidine and thiazole ring moieties that are condensed to form thiamin monophosphate (ThMP), which is subsequently converted to the active form thiamin diphosphate (ThDP). In plants, both thiazole and pyrimidine moieties are synthesized in plastids (Gerdes et al., 2012). *THIC*, the gene encoding hydroxymethylpyrimidine phosphate synthase, the initial step in the pyrimidine biosynthesis branch (Raschke et al., 2007), is feedback regulated by ThDP through a highly conserved riboswitch mechanism (Bocobza et al., 2007). Recent studies suggest that diurnal regulation of *THIC* has a major role in integration of central energy metabolism in plant cells (Bocobza et al., 2013). In addition to the primary biosynthetic pathway, plant genomes encode a network of salvage pathway enzymes (Gerdes et al., 2012; Yazdani et al., 2013; Zallot et al., 2013, 2014).

The diversity of strategies that cells have evolved for acquisition of thiamin is exemplified most fully among bacteria, where species that have lost genes for part or all of the *de novo* thiamin biosynthetic pathway are commonplace. In those organisms, one or both branches of the *de novo* pathway may be replaced by transport systems that enable uptake of thiamin precursors from their environment (Rodionov et al., 2002). Combining information about thiamin growth requirements with analysis of clustering of thiamin-related genes in diverse bacterial genomes has enabled comparative genomics strategies for identifying transporter genes (Rodionov et al., 2002). There is potential for applying similar strategies to plant genomes by comparing transcriptomes of cell types that have different capacities for thiamin synthesis or uptake. Whether significant intercellular transport of intermediates such as hydroxyethylthiazole (HET) or hydroxymethylpyrimidine (HMP) may also occur in plants is unknown.

While evidence of extensive intercellular transport of thiamin in plants has been in hand for at least 70 years, the mechanisms and physiological implications of thiamin transport and salvage are still poorly understood. Roots of most plant species require exogenous thiamin for growth in culture (Bonner, 1940). Bonner and Buchman (1938) determined that cultured pea roots required either thiamin or a mixture of thiazole and pyrimidine components, whereas Robbins and Bartley (1937) reported that cultured tomato roots could grow on thiazole alone suggesting that tomato roots have at least a limited capacity for pyrimidine synthesis. Based on analysis of thiamin auxotroph mutants identified in *Arabidopsis* (Redei, 1965), geneticists surmised that import of maternally synthesized thiamin by the developing seed is sufficient to support seed formation (Shimamoto and Nelson, 1981; Goyer, 2010).

Transport of thiamin and derivatives such as thiamin triphosphate have been hypothesized to have a broader signaling role in plants (reviewed in Goyer, 2010). Thiamin is transported basipetally in tomato stems in a manner analogous to auxin (Kruszewski and Jacobs, 1974), while Mozafar and Oertli (1993) showed that thiamin absorbed by soybean roots moved acropetally. Woodward et al. (2010) have shown that while the maize thiamin biosynthetic protein (THI4) homolog identified by the *blade killer* (*thi2)* mutant is required for shoot apical meristem (SAM) maintenance; THI2 is expressed predominantly in leaf primordia surrounding the shoot apical meristem rather than in the meristem proper. The implication is that thiamin or its precursors are synthesized in the surrounding organs and transported to the meristem. By analogy to bacterial systems, it is also conceivable that there are developmental contexts in which the pyrimidine and thiazole branches of the thiamin biosynthetic pathway are differentially regulated producing a division of labor between cell types.

Recent advances in our understanding of the biochemical mechanism of thiazole biosynthesis in fungi and plants suggest a rationale for why extensive intercellular transport of thiamin may have evolved in plants. The energy cost of thiamin synthesis is high in comparison to other vitamin co-factors. Because the sulfur atom in the thiazole ring is derived from a cysteine residue located in the active site of the THI4 protein in a single-turnover-reaction (Chatterjee et al., 2011), THI4 is technically not an enzyme, but rather a reactant that is consumed in the reaction. There is thus far no evidence in plants or yeast for a complementary reaction that would complete the catalytic cycle and reconstitute active THI4 protein following product release. Evidently synthesis of a single thiazole molecule entails synthesis and eventual degradation of a 320 amino acid polypeptide - a cost of hundreds of ATP equivalents per molecule. The energy cost of thiazole synthesis can be conceptualized as being roughly equivalent to appending a virtual 35 kd subunit to every enzyme that requires a thiamin co-factor. Because the contribution of thiamin to total biomass is very small, the energy cost of thiazole synthesis to a plant overall is still modest, but it is plausibly a significant constraint for non-photosynthetic cells such as shoot and root meristem cells and pollen that have a high-demand for thiamin dependent energy metabolism. Curiously however, in yeasts that lack ThiC as well as the riboswitch feedback mechanism, both thiazole and pyrimidine biosynthetic pathways are controlled by single-turnover reactions (Lai et al., 2012). This suggests that the single-turnover mechanism may have evolved independently in both branches of the thiamine biosynthetic pathway as a mechanism of coordinating thiazole and pyrimidine biosynthesis (Lai et al., 2012). Conceivably, by tying the rate of thiazole synthesis to synthesis of a single-turnover protein is coupled more directly to rates of transcription and translation in plant cells.

Here we analyze and compare the distributions of thiamin biosynthetic capacity in maize and *Arabidopsis* tissues using available transcriptomics data, and we confirm key patterns observed in maize by quantitative real-time reverse transcription polymerase chain reaction (qRT-PCR). The expression of pyrimidine (THIC) and thiazole (THI4) biosynthetic genes is highly correlated in all *Arabidopsis* organs except during embryo maturation where THI4 expression is maintained while THIC expression declines. Divergent expression of THIC and THI4 is more pronounced in maize. We identify at least six developmental contexts in which metabolically active, non-photosynthetic organs exhibit low expression of one or both branches of the *de novo* thiamin biosynthetic pathway indicating a dependence on inter-cellular transport of thiamin and/or thiamin precursors. Neither the thiazole nor pyrimidine branches of the pathway are expressed in developing pollen indicating a dependence on import of thiamin from surrounding floral and inflorescence organs. Consistent with that hypothesis, organs of the male inflorescence and flowers are shown to have high relative expression of the thiamin biosynthetic pathway and thiamin contents that are comparable to leaves. By contrast, divergent patterns of THIC and THI4 expression occur in SAM, embryo, endosperm, and root-tips suggesting that these sink organs acquire significant amounts of thiamin via salvage pathways. In root and shoot meristems, expression of THIC in the absence of THI4 indicates a capacity for thiamin synthesis via salvage of thiazole, whereas the opposite pattern obtains in embryo and endosperm implying that seed storage organs are poised for pyrimidine salvage. Finally, we test the feasibility of tracking thiamin synthesis and transport by stable isotope labeling *in vivo* and determine an upper limit for the rate of *de novo* thiamin biosynthesis in excised maize leaf segments.

## Results and discussion

### Distribution of thiamin biosynthetic capacities in organs of *Arabidopsis* and maize

**Figure 1:**
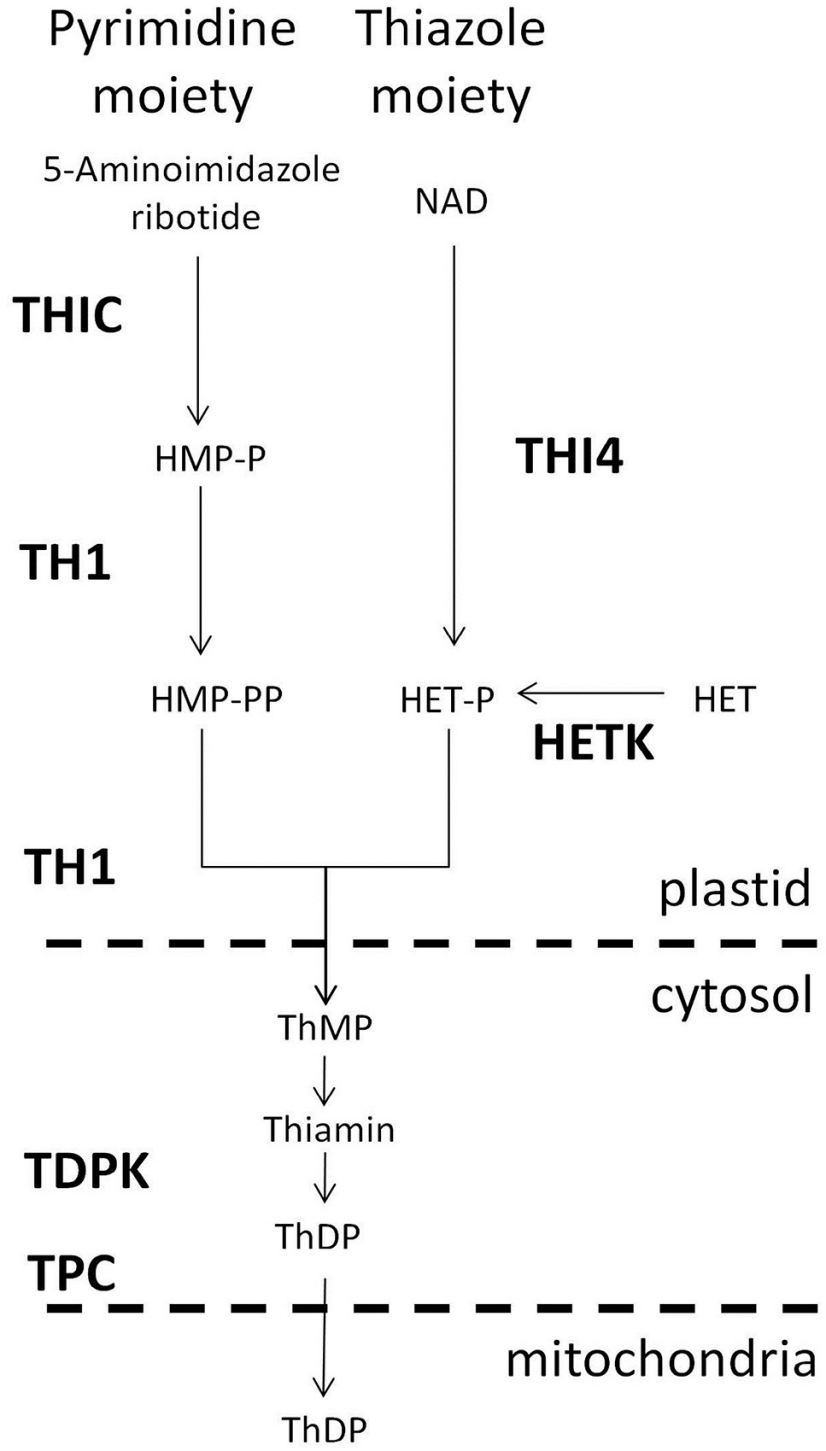
**Thiamin biosynthesis pathway in plants**. Abbreviations (bold) used for enzyme and transport functions assigned to genes of Arabidopsis and maize (Table 1): THIC, hydroxymethylpyrimidine phosphate synthase; THI4, thiazole biosynthetic protein; TH1, hydroxymethylpyrimidine phosphate kinase/hydroxymethylpyrimidine kinase/thiamin-phosphate pyrophosphorylase (dual function enzyme); HETK, hydroxyethylthiazole kinase; TDPK, thiamin diphosphokinase; TPC, mitochondrial thiamin diphosphate carrier. Pathway intermediates and products include HMP-P, hydroxymethylpyrimidine phosphate; HMP-PP, hydroxymethylpyrimidine diphosphate; thiamin; ThDP, thiamin diphosphate; HET, hydroxyethylthiazole; HET-P, hydroxyethylthiazole phosphate. Dashed horizontal lines separate plastidial, cytosolic and mitochondrial compartments of the cell.

In order to explore the distribution of thiamin biosynthetic capacity among plant organs, we took advantage of the thorough annotation of maize and *Arabidopsis* thiamin pathway genes provided in the PlantSEED (Figure 1; Gerdes et al., 2012). To minimize confusion due to inconsistency of genes names used in various species, for this study we have adopted the abbreviations listed in Table 1. In the *de novo* biosynthesis pathway, THIC and TH1 are encoded by single genes in maize and *Arabidopsis*, whereas two genes encode the thiamin diphospokinase (TDPK) and mitochondrial thiamin diphospate transporter (TPC) activities in both species (Zallot et al., 2013). *Arabidopsis* has a single THI4 gene, while the maize genome contains two THI4 paralogs (*Thi1* and *Thi2;* Belanger et al., 1995; Woodward et al., 2010). In addition, we included in our analysis several salvage pathway genes (HETK, Goyer et al., 2013; NUDIX, Yazdani et al., 2013), and two genes of the TenA family that are implicated in salvage of the pyrimidine moiety (Zallot et al., 2014), as well as a COG0212 gene of unknown function implicated in thiamin metabolism by associations in plants and bacteria (Pribat et al., 2011).

**Table 1:** **Thiamin biosynthetic pathway genes of maize and *Arabidopsis***.

**Abbreviation**	**Biochemical function**	**Maize gene**	***Arabidopsis* gene**
THI4	Thiazole biosynthetic protein	Thi1, GRMZM2G018375	TZ, At5g54770
		Thi2, GRMZM2G074097	
THIC	Hydroxymethylpyrimidine phosphate synthase	GRMZM2G027663	PY, At2g29630
TDPK	Thiamin diphosphokinase	GRMZM2G055458	TDPK1, At1g02880
		GRMZM5G864815	TDPK2, At2g44750
TH1	Hydroxymethylpyrimidine phosphate kinase/hydroxymethylpyrimidine kinase/thiamin-phosphate pyrophosphorylase	GRMZM2G401934	TH1, At1g22940
HETK	Hydroxyethylthiazole kinase	GRMZM2G094558	At3g24030
TENA1	Thiaminase II	GRMZM2G078283	At5g32470
		GRMZM2G148896	
TENA2	Thiaminase II homolog	GRMZM2G080501	At3g16990
NUDIX	Oxy-, oxi- thiamin phosphatase	GRMZM2G031461	NUDT20, At5g19460
			NUDT24, At5g19470
COG0212	Thiamin associated 5-formyltetrahydrofolate cycloligase paralog	GRMZM2G001904	At1g76730
TPC	Mitochondrial thiamin diphosphate transporter	GRMZM2G124911	TPC1, At3g21390
		GRMZM2G118515	TPC2, At5g48970

**Figure 2:**
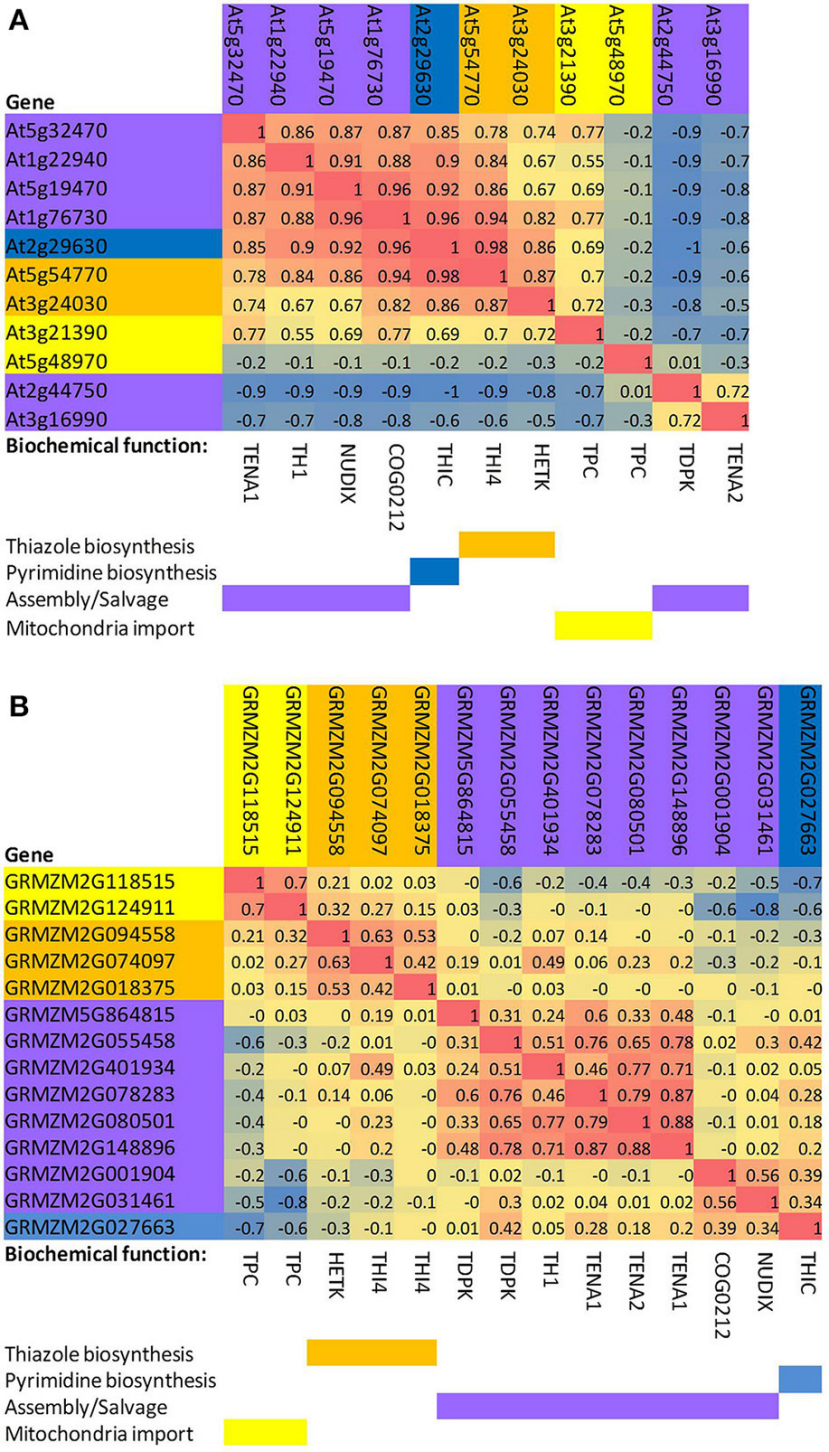
**Correlated expression of thiazole, pyrimidine, transport, and salvage genes of the thiamin biosynthesis pathway in *Arabidopsis* and maize**. **(A)** *Arabidopsis*. A profile for each gene was calculated based on Pearson rank-order correlations with other thiamin pathway genes in the AtGeneExpress development dataset (Schmid et al., 2005). Genes were then clustered based on the matrix of pairwise correlations (R2 values) among the gene profiles. **(B)** Maize. A profile for each gene was constructed by calculating Pearson rank-order correlations with other thiamin pathway genes in the QTELLER transcriptome dataset (see Methods). Genes were then clustered based on the matrix of pairwise correlations (R2 values) among the gene profiles. TPC, mitochondrial thiamin diphosphate transporter; THI4, thiazole biosynthesis protein; HETK, hydroxyethyl thiazole kinase;. TDPK, thiamin diphosphokinase paralogs; TH1, hydroxymethylpyrimidine phosphate kinase/hydroxymethylpyrimidine kinase/thiamin-phosphate pyrophosphorylase, dual function protein; COG0212, putative thiamin related 5-formyltetrahydrofolate cycloligase-like protein, function unknown; TENA1 and TENA2, thiaminase II paralogs; NUDIX, putative thiamin related NUDIX type hydrolase; THIC, hydroxymethylpyrimidine phosphate synthase.

To examine and compare coordinate regulation of the thiamin pathway in diverse organs of *Arabidopsis* and maize, we performed a rank correlation analysis of thiamin gene expression based on the AtGeneExpress development series (Schmid et al., 2005) for *Arabidopsis* (Figure 2A) and the QTELLER.org transcriptome database for maize (Figure 2B), respectively. In *Arabidopsis*, the genes encoding THI4 and THIC have highly correlated expression through development and indeed show the highest correlation (*R*^2^ = 0.98) of any pair of genes in the thiamin pathway. By contrast, THI4 and THIC genes exhibit divergent regulation in maize where they occupy distinct clusters in the correlation matrix (Figure 2B). While this difference may reflect biases in the organs represented in the *Arabidopsis* and maize datasets as well as fundamental differences in the regulation of the thiamin pathway in the two species, the propensity of species to exhibit different biases increases the power of comparative analysis much as it has in bacterial genomics. Hence, the maize system is potentially a source of new insights into the regulation of thiamin biosynthesis and transport in plants.

Interestingly, other patterns such as clustering of THIC with the NUDIX thiamin-phosphatase (Goyer et al., 2013) and COG0212 genes, and the close correlation between THI4 and HETK are conserved in *Arabidopsis* and maize. Co-regulation of HETK with *de novo* thiazole biosynthetic capacity is consistent with HETK functioning in salvage of the thiazole moiety (Yazdani et al., 2013) under conditions where thiazole may be limiting. Because the NUDIX thiamin-phosphatase, which is implicated in de-phosphorylation of toxic oxygenated forms of thiamin diphosphate (Goyer et al., 2013), operates on molecules containing defects in either ring moiety, its activity is not uniquely associated with the pyrimidine branch of the pathway. Rather, its close association with THIC may reflect the role of THIC as a key control point in the biosynthetic pathway overall (Bocobza et al., 2013). While the specific biochemical function of COG0212 is unknown (Pribat et al., 2011); the strong association with THIC suggests that it functions either in salvage of pyrimidine or that it has an activity related to the NUDIX mediated thiamin-repair pathway (Goyer et al., 2013).

**Figure 3:**
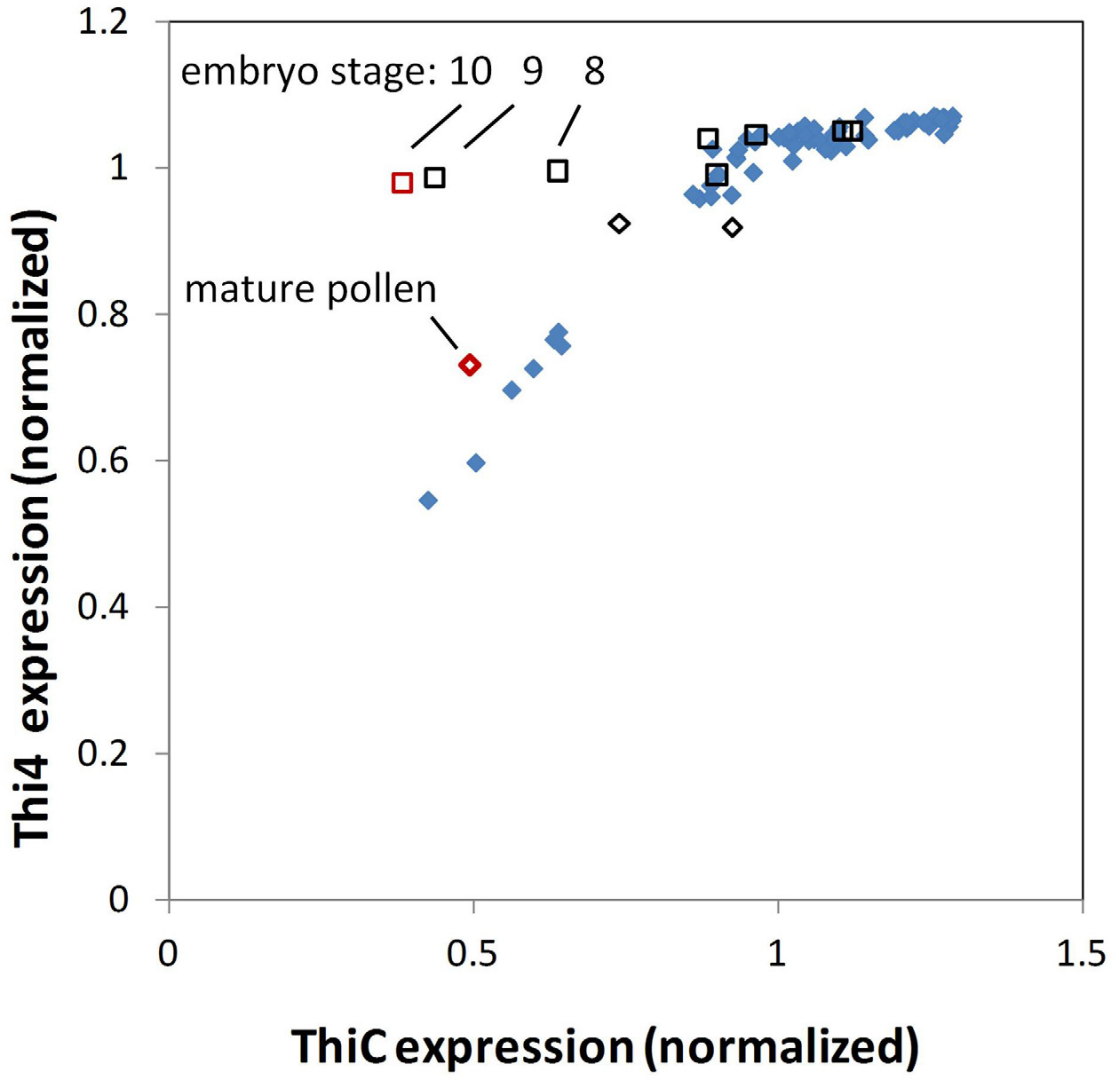
**THIC and THI4 expression in Arabidopsis tissues**. AtGeneExpress values (log2) for THIC and THI4 were normalized to the respective overall mean of each gene. Relative THI4 expression is strongly correlated with THIC expression except in the later stages of seed development (open squares, red is mature stage) where THIC expression declines sharply during embryo maturation while THI4 expression remains high. Seed at earlier stages (black open squares), stamens containing developing pollen (black open diamonds) and mature pollen (red open diamond) conform to the pattern of proportional expression of THI4 and THIC observed in other tissues (blue solid diamonds).

**Figure 4:**
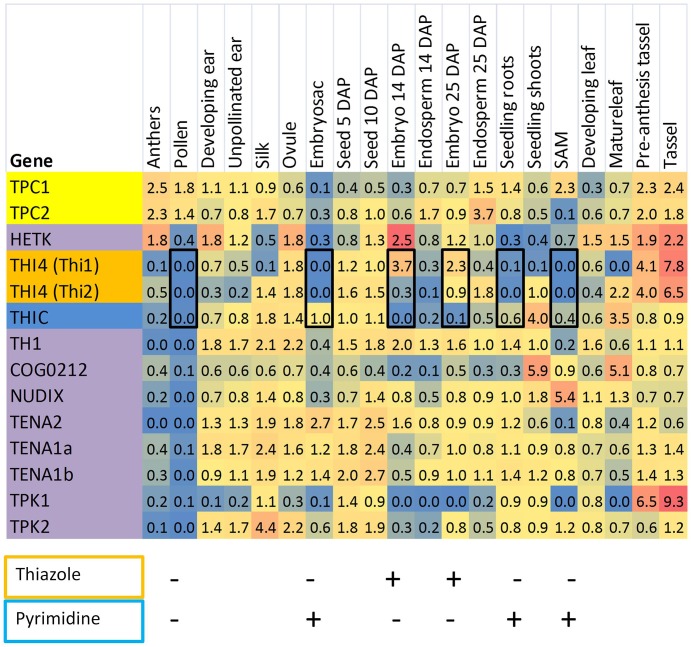
**Distribution of thiamin biosynthetic capacities of maize tissues inferred by transcriptomics**. A heat map was constructed from transcriptome datasets compiled at QTELLER.org by normalizing FPKM values to the mean for each gene across all tissues. Shades of red indicate high expression relative to the mean and blue shading denotes low relative expression. On the left panel, genes encoding thiazole biosynthesis functions are highlighted gold, and the pyrimidine synthetic gene (THIC) is colored blue. Other genes involved in thiamin assembly and salvage are colored purple, and mitochondrial transporter genes are highlighted in yellow. Black rectangles highlight six developmental contexts that show either low expression or divergent expression of genes encoding THIC and THI4. For these cases, qualitative patterns of thiazole (outlined in gold) and pyrimidine (outlined in blue) biosynthetic capacity are summarized as being present (+) or low/absent (–). SAM, shoot apical meristem.

The close correlation of THI4 and THIC expression in most *Arabidopsis* organs is depicted in Figure 3. A striking exception occurs during later stages of embryo development where THIC expression declines sharply while relative THI4 expression remains high. By comparison, the distribution of thiamin gene expression in the maize QTELLER dataset (Figure 4) revealed at least five developmental contexts where expression levels of THIC and THI4 diverge, and a sixth context, mature pollen, where gene expression for the thiamin *de novo* pathway overall is very low. As is the case for the *Arabidopsis* embryo, the relative expression of maize THI4 paralogs *Thi1* and *Thi2* is high compared to relative THIC expression in the developing embryo and endosperm (14 days after pollination, DAP), whereas in the shoot apical meristem (SAM) and in seedling roots the opposite pattern obtains in which THIC expression is high relative to THI4. Consistent with results of Woodward et al. (2010), the *Thi1* and *Thi2* paralogs exhibit divergent expression in 14 DAP endosperm, silks, seedlings and mature leaves, a pattern indicative of partial sub-functionalization at the level of gene regulation. Woodward et al. (2010) concluded that expression of *Thi2* in leaf primordia surrounding the SAM is essential for maintenance of the SAM in maize implying that thiamin is transported to the SAM from surrounding organs. However, the transcriptome profile (Figure 4) indicated that in contrast to the THI4 paralogs, THIC is expressed in the SAM suggesting that the *de novo* pyrimidine biosynthesis pathway is potentially active in meristem cells which would enable thiamin synthesis via salvage of thiazole. In that case, it is plausible that the SAM is sustained at least partly by import of thiazole intermediates (e.g., HET) from surrounding leaves as well as thiamin.

To extend and confirm the results of the transcriptome analysis, we focused on three developmental contexts that exhibit distinctive patterns of thiamin pathway regulation: developing pollen where the *de novo* thiamin biosynthetic pathway is apparently inactive; and the developing seed and roots, respectively, that exhibit reciprocal patterns of regulation of the thiazole and pyrimidine branches of the thiamin biosynthetic pathway.

### Pollen depends on sporophytic sources of thiamin

The transcriptome analysis indicated that most thiamin biosynthetic genes are inactive in mature maize pollen (Figure 4). Expression of those genes was similarly low in fully developed anthers (Davidson et al., 2011), the chief living constituent of which is mature pollen. By contrast, expression of mitochondrial thiamin transporter genes, which are thought to be essential for thiamin dependent metabolism in living cells (Zallot et al., 2013), as well as the HETK gene required for thiazole salvage, was comparatively high in both anthers and pollen consistent with the expected metabolic activity of pollen. Because pollen is expected to have a high demand for thiamin, these results raise the question of how and when pollen acquires its thiamin. In sharp contrast to mature pollen, male inflorescence (tassel) as well as silks, the pollen receptive organ of the female inflorescence, showed high relative expression of *de novo* thiamin biosynthetic pathway genes. Based on these results, we hypothesized that thiamin contained in pollen may be (1) imported primarily from surrounding organs of the male inflorescence and/or (2) acquired from silks at the time of pollen germination.

While the transcriptome of mature pollen suggests that it acquires thiamin from an external source, we could not rule out the possibility that thiamin biosynthesis is active in developing pollen grains and subsequently down-regulated during pollen maturation. We noticed, for example, that key genes in the starch biosynthetic pathway including the *Wx1* starch synthase (data not shown) also showed very low expression in the mature pollen dataset (Davidson et al., 2011), whereas *Wx1* is known from genetic evidence to function in starch synthesis during pollen development (Nelson, 1962). Thus, we concluded that with respect to starch biosynthesis at least, the transcriptome of mature pollen is most likely not representative of developing pollen.

**Figure 5:**
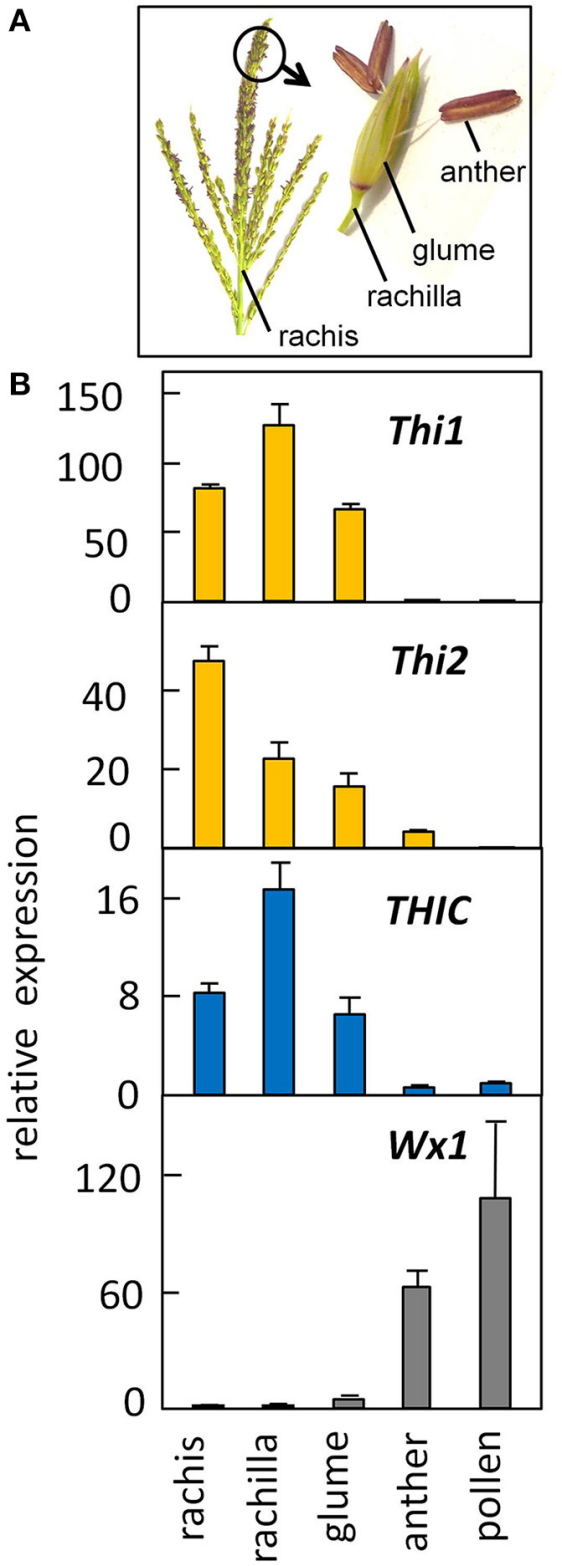
**Low expression of the thiamine biosynthetic pathway in developing pollen contrasts with high expression in surrounding floral organs**. **(A)** Structure of the male inflorescence of maize (image, J. Saunders; art, K. E. Koch). **(B)** Gene expression in floral organs and developing pollen. RNA was quantified by qRT-PCR (see Methods). Late-binucleate stage pollen was isolated from developmentally staged anthers as described by Wen and Chase (1999). The *Wx1* starch synthase was measured as a positive control for pollen gene expression. Error bars are standard error of the mean, *n* = 3.

To determine whether thiamin biosynthetic genes are expressed in developing pollen, we analyzed thiamin gene expression in pollen grains isolated during the starch-filling stage of pollen development. Figure 5 shows quantification of *Thi1*, *Thi2* and THIC expression in the organs of the male inflorescence (Figure 5A); in binucleate-stage developing pollen; and in pre-anthesis stage anthers. The qRT-PCR results confirmed that expression of thiazole and pyrimidine biosynthetic genes is very low in both anthers and developing pollen in contrast to the high expression observed in rachis (stem of the male inflorescence) and rachilla and glume organs of the male flower. As a positive control, we assayed expression of *Wx1*, which is known to function in developing pollen (Nelson, 1962). As expected, developing anthers and pollen exhibited high relative expression of *Wx1* supporting our conjecture that the starch biosynthetic pathway is down-regulated during pollen maturation most likely immediately prior to anthesis. By contrast, the *de novo* thiamin biosynthetic pathway is inactive in starch-filling pollen as well as mature pollen.

**Figure 6:**
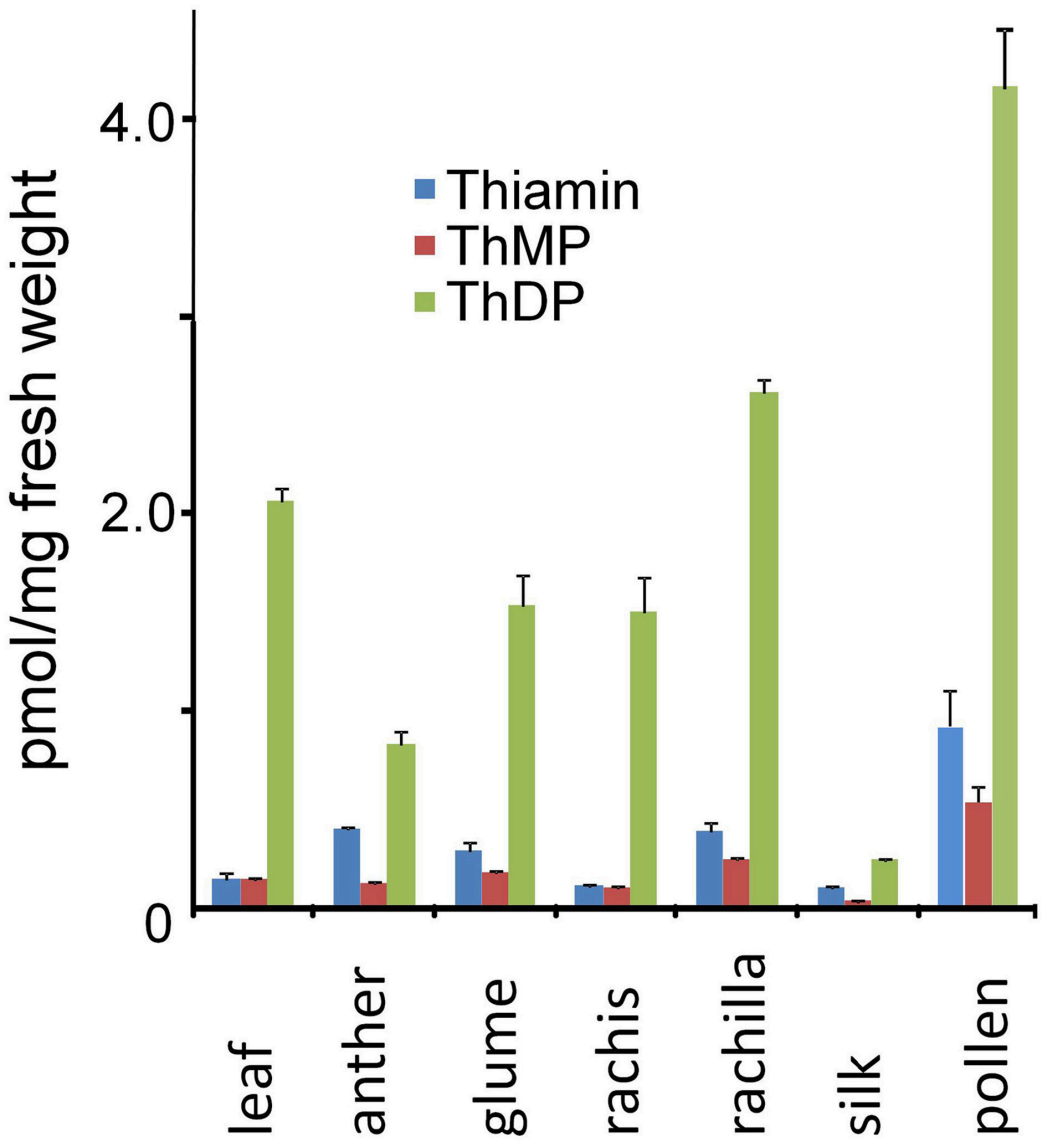
**Thiamin and thiamin phosphate ester contents of maize leaves, pollen, inflorescence and floral organs**. Thiamin content (pmol/mg of FW tissue) was determined as described in Methods. Pre-anthesis anthers were sampled 1–2 days prior to expected anthesis. Bars: Standard error of the mean, *n* = 3.

As shown in Figure 6, consistent with high relative expression of the thiamin biosynthetic pathway, thiamin and thiamin phosphate contents of rachilla, glume and rachis organs of the male inflorescence (Figure 5A) were comparable to or greater than that of leaves. The total thiamin and thiamin phosphate contents of pre-anthesis stage anthers was about 50% of leaf levels consistent with substantial accumulation of thiamin during pollen development. Remarkably, on a fresh weight basis the thiamin and thiamin phosphate contents of mature pollen collected at anthesis was about 5-fold greater than in anthers sampled 1–2 days prior to anthesis. While it is conceivable that rapid accumulation of thiamin forms occurred in the final days of pollen maturation, our interpretation of the difference between anthers and pollen is tempered by two undetermined factors affecting fresh weight; (1) the dilutive effect of non-living sporophytic tissues of the anther that most likely contribute very little to thiamin content of the organ and (2) the concentrating effect of water loss from maize pollen as it is shed during anthesis. Consistent with these effects, the distributions of thiamin and ThDP in pollen and pre-anthesis anthers were qualitatively similar. In any case, while the detailed time course of thiamin accumulation in pollen is not yet resolved, it is clear that mature pollen acquires comparatively high thiamin content prior to anthesis. By contrast, in spite of having high relative expression of the biosynthetic pathway, silks had comparatively low content of thiamin forms indicating that pollen is unlikely to be supplied with thiamin from maternal tissues during germination. It remains possible that silks are biosynthetically active and export thiamin to the subtending ovary during floral development.

Together, these results support the hypothesis that developing pollen is dependent on import of thiamin forms and/or thiamin precursors that are synthesized in surrounding organs of the male inflorescence and flower. Our analyses of mRNA levels do not rule out presence of thiamin biosynthetic enzymes in developing pollen. In addition to analyses of protein levels, direct measurements of thiamin transport into pollen will be needed to obtain a definitive test of the thiamin import hypothesis.

### Divergent regulation of THIC and THI4 in roots and developing embryo and endosperm

**Figure 7:**
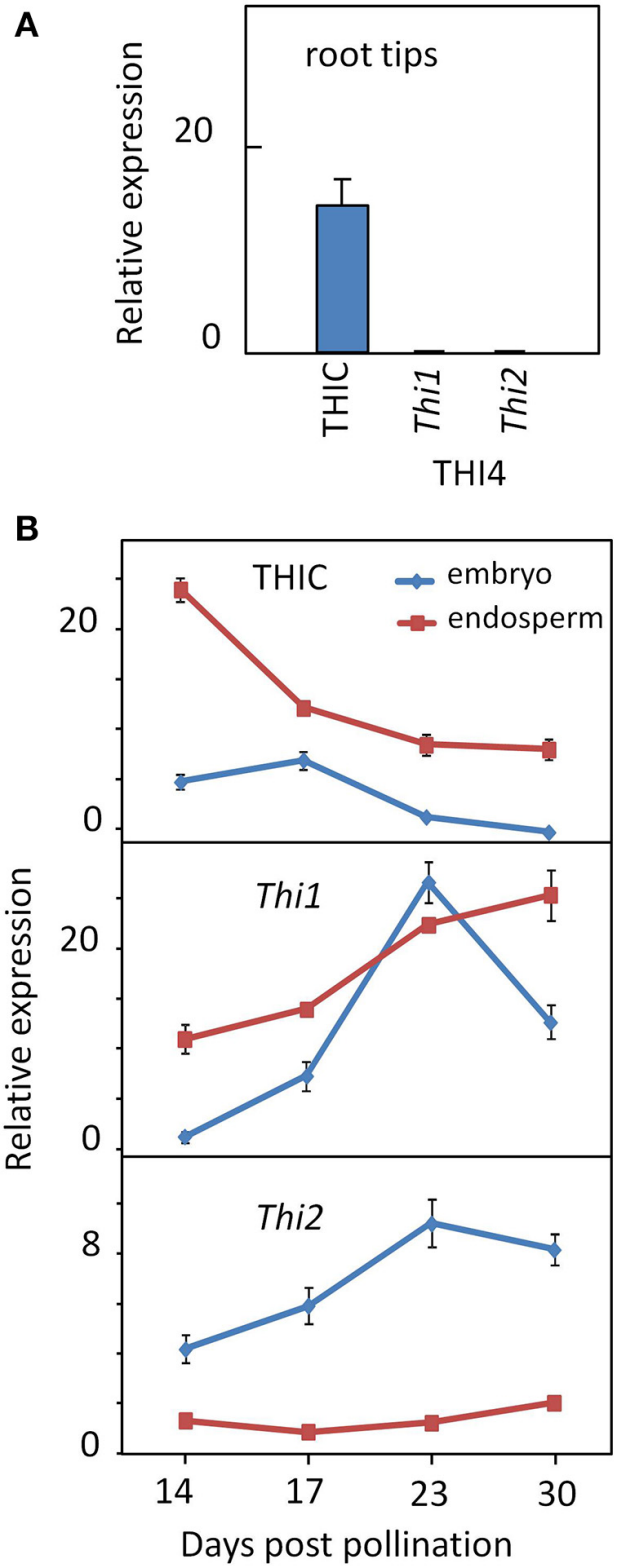
**Expression of thiamin biosynthetic genes in root tips and seed tissues**. **(A)** THIC expression in absence of THI4 in maize root tips. **(B)** Divergent regulation of THIC and THI4 genes in developing embryo and endosperm. Expression of THIC, *Thi1* and *Thi2* genes in root tips and developing embryo and endosperm was assayed by qRT-PCR (Guan et al., 2012; see Methods). Trace amounts of *Thi1* and *Thi2* transcripts were detected in root tips. Error bars are standard error of the mean (*n* = 3).

To confirm the patterns of divergent regulation of thiazole and pyrimidine biosynthetic capacities inferred from the transcriptome analysis (Figure 4), we quantified THIC and THI4 gene expression in seedling root tips and developing embryo and endosperm using qRT-PCR (Figure 7). Consistent with the RNA-seq data, THIC transcript levels were relatively low, but clearly present in root tips, whereas neither of the THI4 paralogs was expressed at detectable levels (Figure 7A). This contrasts with the low, but balanced expression of THIC and THI4 observed in *Arabidopsis* roots (Figure 3). These results imply that maize root tips lack capacity for complete *de novo* synthesis of thiamin, but have at least a potential for synthesis of thiamin via salvage of thiazole intermediates. This may include thiazole generated *in situ* through metabolism and turnover of imported thiamin and/or thiazole transported to roots from elsewhere in the plant - or more speculatively, taken up from the rhizosphere where it is produced and secreted by microbes. Early studies indicate that the capacity of roots to synthesize thiamin from exogenously supplied thiazole varies among species (Robbins and Bartley, 1937; Bonner and Buchman, 1938; Bonner, 1940). The presence of THIC transcript in the absence of THI4 expression observed in roots is similar to the situation in the SAM noted above, suggesting that this is a shared feature of meristematic tissues.

By contrast, the opposite pattern occurred in the maize seed, where one or both of the paralogs encoding THI4 were active in embryo and endosperm while relative expression of THIC was low. Belanger et al. (1995) have previously shown that *Thi1* is highly expressed in the developing maize embryo. A developmental time course of THIC and THI4 transcript levels (Figure 7B), confirmed that THIC expression in both embryo and endosperm declined late in development while relative expression of both THI4 paralogs (*Thi1* and *Thi2*) increased. While the *Thi1* paralog of THI4 was active in both embryo and endosperm, *Thi2* was expressed preferentially in the embryo. Overall, these results confirm that divergent regulation of THI4 and THIC during seed development is conserved in maize and *Arabidopsis* in spite of the manifold differences in embryo and endosperm development in the two organisms. In contrast to the *Arabidopsis* embryo, for example, the maize embryo lacks photosynthetic capacity throughout its development. While divergent expression of the thiazole and pyrimidine biosynthetic capacities is formally consistent with the possibility that seeds have a capacity for synthesis of thiamin via salvage of a pyrimidine intermediate (e.g., HMP) imported from maternal tissues, genetic studies in *Arabidopsis* indicate that expression of neither THIC nor THI4 in the seed is essential for seed formation (Redei, 1965; Goyer, 2010). Developing *Arabidopsis* seeds may acquire as much as 90% of their thiamin content from maternal sources (Goyer, 2010) and developing maize kernels actively import thiamin from maternal tissues (Shimamoto and Nelson, 1981). Plausibly, an ample maternal supply of thiamin could cause down-regulation of THIC (Bocobza et al., 2007, 2013), but that does not explain why high expression of THI4 in the developing seed is conserved. One possibility is that thiazole biosynthetic capacity is maintained in the seed in order to support salvage of the pyrimidine component from damaged thiamin produced in metabolically highly active seed tissues. There is evidence that thiamin is degraded at a significant rate during catalysis of thiamin requiring enzymes (Goyer, 2010), and although the mechanism and products of degradation are poorly understood it is clear that the pyrimidine moiety is generally more stable than the thiazole moeity and hence more likely to be salvaged (Zallot et al., 2014).

**Figure 8:**
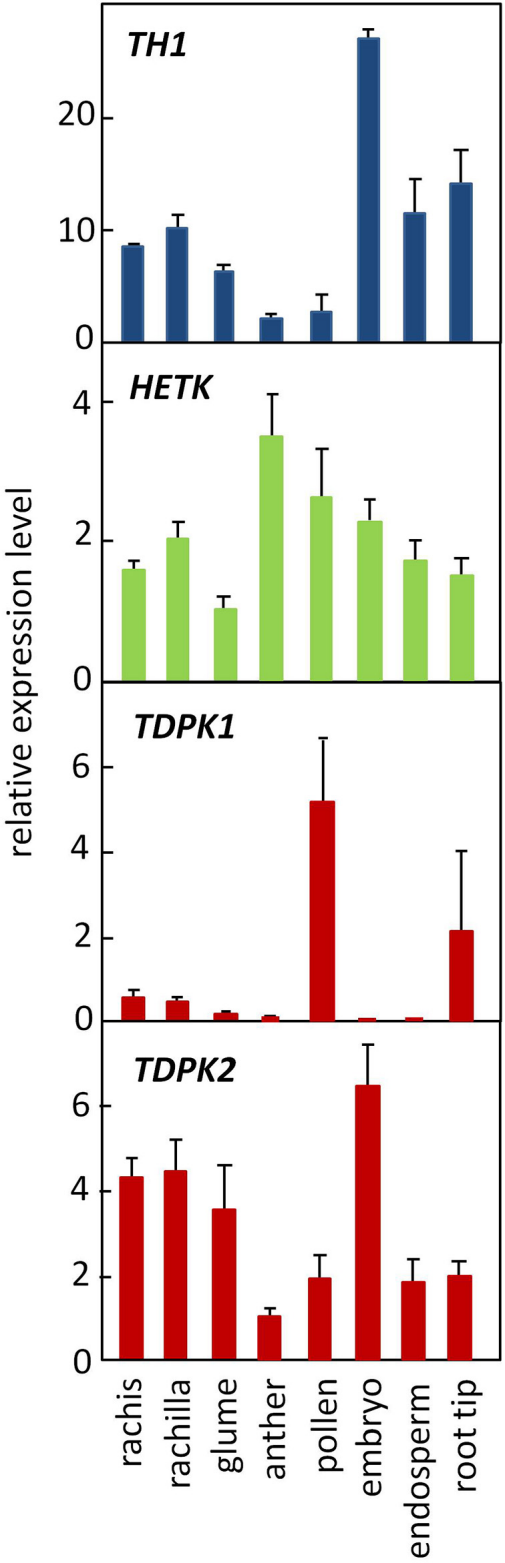
**Genes encoding thiamin assembly and salvage steps are ubiquitously expressed in maize**. TH1 (blue), TDPK1 and TDPK2 (red) function in primary synthesis and thiamin salvage pathways. HETK functions in thiazole salvage. Error bars are standard error of the mean (*n* = 3).

The opposing patterns of THIC and THI4 expression observed in meristems vs. filial organs of the seed raises the possibility that the relative flux through thiazole and pyrimidine salvage pathways, respectively, may differ in meristems and seed organs. Consistent with that hypothesis, relative expression of TH1, which functions in pyrimidine salvage as well as *de novo* synthesis, is high in the embryo compared to roots (Figure 8). However, expression of HETK was comparable in seed organs and root tips indicating a significant capacity for thiazole salvage in both contexts. As noted above, HETK clusters more strongly with THI4 than it does with TH1 or THIC (Figure 2B) suggesting it is broadly correlated with thiazole demand. By contrast, in the organs assayed in Figure 8, and in organs overall (Figure 4), TDPK2 correlated more strongly with TH1 and THIC than with the thiazole biosynthetic genes (*Thi1* and *Thi2*). This pattern is consistent with the rate of pyrimidine biosynthesis being key to the overall control of thiamin biosynthesis (Bocobza et al., 2007, 2013). While the full metabolic implications of independent regulation of the thiazole biosynthetic pathway in maize organs remain to be elucidated, studies in rice implicate induction of THI4 in disease resistance (Wang et al., 2006).

Together the gene expression data delineate three distinctive patterns of thiamin pathway regulation in heterotrophic maize organs that parallel strategies for thiamin production that have evolved in bacteria: (1) complete dependence on external sources of thiamin (developing pollen), (2) capacity for *de novo* thiazole biosynthesis coupled with pyrimidine salvage (seed tissues), and (3) capacity for *de novo* pyrimidine biosynthesis coupled with thiazole salvage (root and shoot meristems). This division of labor implies that the intercellular networks comprised of thiamin-related metabolic dependencies, transporters and signaling interactions will vary fundamentally depending on the developmental context. Direct tests of this hypothesis will ultimately require means of tracking thiamin synthesis and transport at the cellular level.

### Feasibility of measuring thiamin biosynthesis by stable isotope labeling in leaf explants

**Figure 9:**
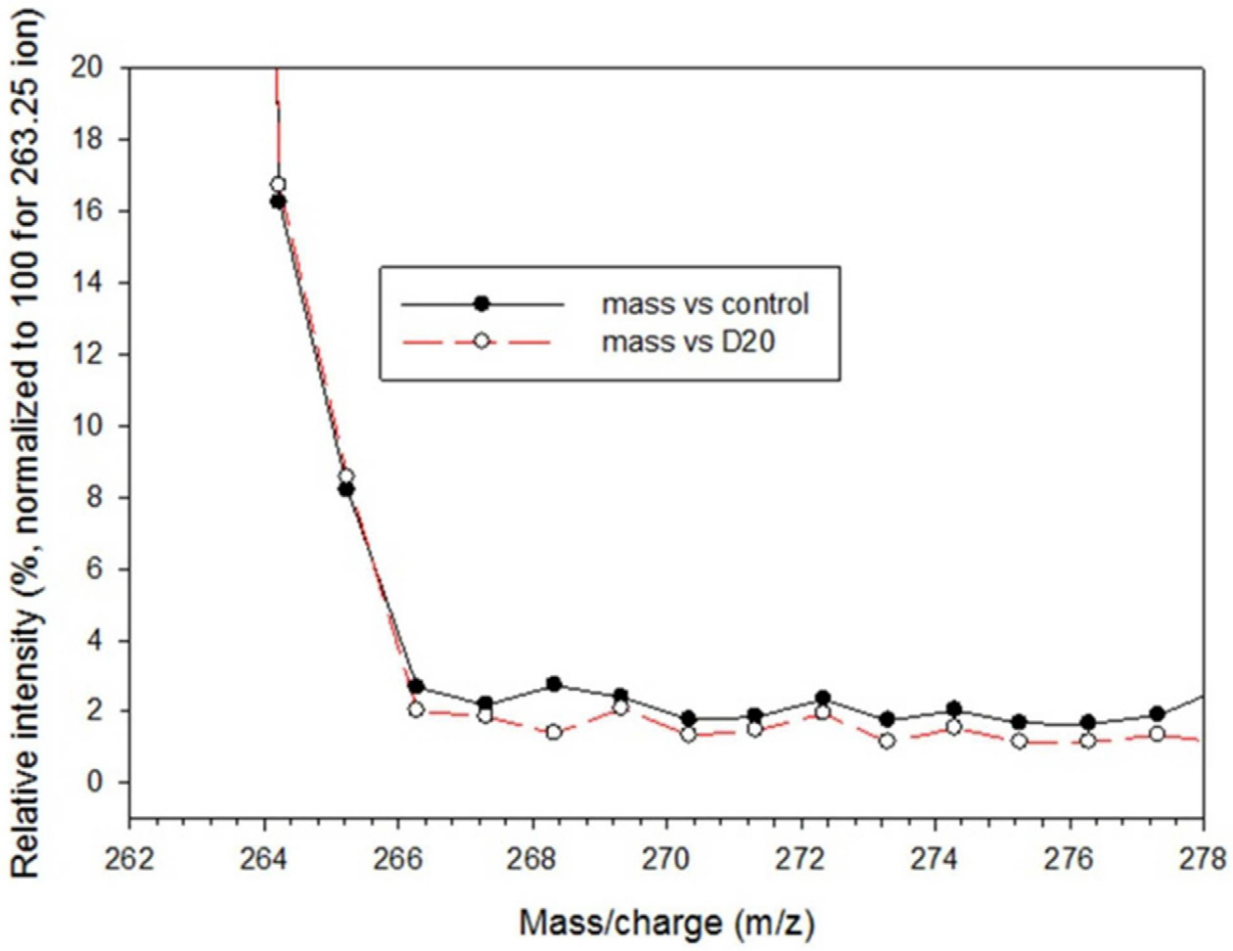
**Mass spectrometric analysis of thiamin vitamers extracted from D_2_O labeled maize leaf slices and analyzed as thiochrome**. Leaf slices were floated on water containing 30% D_2_O and incubated in the light for 24 h. Thiamin and its phosphates were extracted and converted to thiochrome. A full scan was collected from *m/z* 100–600 and a zoom scan was collected from *m/z* 260–270 to evaluate isotopic enrichment. Intensity values are normalized relative to the molecular ion signal of 263.25 (set equal to 100). The profile of isotopolog masses up to M+13 is shown to allow detection of deuterium incorporation over a complex background due to natural stable isotope abundances of ^13^C, ^17^O, ^18^O, ^15^N, and ^34^S. Data points are means of two independent samples.

*In vivo* isotope labeling experiments are a potentially powerful approach to testing hypotheses for thiamin biosynthesis and transport based on transcript analyses. Direct measurements for rates of thiamin biosynthesis in plant organs are currently lacking. To estimate an expected rate for leaf tissue during the vegetative phase of development, we reasoned that at a minimum the average rate of thiamin synthesis must be sufficient to keep pace with the overall daily rate of biomass accruing due to plant growth assuming that the thiamin contents of leaves and other tissues are relatively constant throughout development. In that case, the relative net rate of thiamin synthesis in leaves should match the relative growth rate for biomass, which for maize is about 10% per day during the vegetative growth phase (Pérez-Leroux and Long, 1994). A rate of thiamin synthesis equivalent to 10% of the existing pool every 24 h should be readily detected by stable isotope incorporation and mass spectrometry. Studies of the THIC reaction mechanism indicate that at least one hydrogen atom derived from water is incorporated into a non-exchangeable position of pyrimidine ring (Lawhorn et al., 2004) indicating that it should be possible to label *de novo* synthesized thiamin in plant tissues incubated with D_2_O. To test the feasibility of stable isotope labeling of thiamin, maize leaf segments were incubated by floatation on 30% D_2_O enriched water in the light for 24 h. Thiamin and its phosphates were then extracted from D_2_O labeled and H_2_O treated control leaf samples, dephosphorylated, and converted to thiochrome, which was analyzed by liquid chromatography/mass spectrometry. Quantitative comparison of isotopolog profiles of thiochrome from D_2_O treated and control samples failed to detect incorporation of deuterium into thiamin (Figure 9). Allowing for experimental variation, we estimate conservatively that the highest relative incorporation rate that could have escaped detection in this experiment is about 3% per day, a rate that is still several-fold lower than the minimum rate predicted by the relative growth requirement. While identifying the cause of this discrepancy will require further investigation, one plausible explanation is rapid in-activation of thiamin biosynthesis in leaf segments following excision. Rapid in-activation of thiamin biosynthesis in response to injury would plausibly be an important defense against pathogen invasion. In any case, it is evident that further progress toward measurement of *in vivo* thiamin biosynthesis and transport rates will require exploration of new methods including labeling of intact organs or plants.

## Methods

### Transcriptome analyses

Transcriptome analyses of the *Arabidopsis* thiamin biosynthetic genes were based on the 79 tissue development series of AtGeneExpress (Schmid et al., 2005). Transcriptome analyses of maize thiamin genes were based on 22 published RNA-seq datasets assembled in the QTELLER.org database. The organs included mature anthers, developing ear, ear, embryo 25 days after pollination (DAP), endosperm 25 DAP, ovule, mature pollen, developing tassel, mature tassel, seed 5 DAP, seed 10 DAP, silk, and seedling leaves (Davidson et al., 2011); shoot apical meristem (Jia et al., 2009), developing leaf and mature leaf (Li et al., 2010); seedling roots and seedling shoots (Wang et al., 2009); embryo 14 DAP and endosperm 14 DAP (Waters et al., 2011); and bundle sheath and mesophyll (Chang et al., 2012).

To cluster genes in the thiamin pathway of *Arabidopsis* and maize, a profile was first constructed for each thiamin gene by calculating its Pearson rank correlation (R-project.org) pair-wise with each gene in the pathway based on expression values in the AtGeneExpress (log2 transformed values, Schmid et al., 2005) and QTELLER.org databases, respectively. Hierarchical clustering was then performed on a correlation matrix constructed from the gene profiles using the dist and hclust functions in R (R-project.org). Rows and columns in the heat maps are arranged according to each genes position in the cluster tree with self-correlations occurring on the diagonal.

An expression heat map for maize thiamin genes was constructed by normalizing the FPKM values for each organ to the mean value for all organs. The normalized map was colored using the built in conditional formatting tool in Excel (Microsoft, Inc).

### Plant materials and growth conditions

For RNA analyses of seedling tissues, B73 inbred maize seeds were sown in plastic pots filled with Metro-Mix 910 potting mixture (Sun-Gro Inc, Agawam, MA) and grown in a walk-in growth chamber at 24°C with a 16/8-h light/dark cycle. For assays of mature plants, tissue samples were taken from field-grown plants (B73 and W22 inbred) at the University of Florida research farm in Citra, Florida. All tissue samples were frozen in liquid nitrogen immediately and then kept at a −80°C freezer until used. Late-binucleate stage pollen grains were isolated from staged tassels of Mo17 inbred plants by centrifugation through a 70% w/v sucrose density step gradient as described by Wen and Chase (1999).

### Gene expression analysis by quantitative real-time RT-PCR (qRT-PCR)

For expression analysis of maize genes for thiamin biosynthesis, tissues were ground in liquid nitrogen. Total RNA was extracted and purified using the plant RNeasy kit (Qiagen) according to the manufacturer's instructions. A modification for pollen, endosperm and embryo samples was that the ratio of RLT buffer to sample was changed to 2 ml buffer per 20 mg sample. Total RNA was quantified using a NanoDrop 1000 (Thermo Fisher Scientific) instrument, and 5.5 μg of RNA from each sample was treated with the RQ1 RNase-free DNase (Promega). Negative controls without reverse transcriptase added were used to confirm that there was no carryover of genomic DNA. For quantitative real-time PCR (qRT-PCR), a Power SYBR green RNA-to-C*_T_* 1-step kit (Applied Biosystems) was used with an iCycler iQ real-time PCR detection system (Bio-Rad). The *18S rRNA* was used as an internal reference as described in Guan et al. (2012) and following the mathematical model of Pfaffl (2001). Amplification efficiencies of the gene-specific primer pairs (Table 2) ranged between 95 and 105%. However, qRT-PCR results were not adjusted for variation in primer efficiencies, so quantitative comparisons between genes were not attempted.

**Table 2:** **Gene specific primers for qRT-PCR**.

18S rRNA control	Forward:	5′-ATTCTATGGGTGGTGGTGCAT-3′
	Reverse:	5′-TCAAACTTCGCGGCCTAAA-3′
*TH1* (GRMZM2G401934)	Forward:	5′-GTCCACCGACATGTCCGAGAG-3′
	Reverse:	5′-CTACTGCTCCTCCGTAATCAC-3′
*THI1* (GRMZM2G074097)	Forward:	5′-TGCCTTGTTGTTCAATGATGA-3′
	Reverse:	5′-GTGGTGGTGCTATGAACACG-3′
*THI2* (GRMZM2G018375)	Forward:	5′-TGGGACTTTGTTGTTGTTGG-3′
	Reverse:	5′-GTAGCTGTGGCATGGTGCTA-3′
*HETK* (GRMZM2G094558)	Forward:	5′-GCTCTGCCCAGCAGTCGTCAG-3′
	Reverse:	5′-CAGCTTCAATAGCATCAACAGAG-3′
*THIC* (GRMZM2G027663)	Forward:	5′-GATGCAAATGATAGTGCACAG-3′
	Reverse:	5′-ACTCCAACTGTTTCTCCATG-3′
*TDPK1* (GRMZM5G864815)	Forward:	5′-CTTCTGGTCTTACTGAGTCCATATC-3′
	Reverse:	5′-CAACCGCGTCTTCGACGGCATG-3′
*TDPK2* (GRMZM2G055458)	Forward:	5′-TGGATGTGCTTATCATGCTC-3′
	Reverse:	5′-TGAGTCGTCTGTTGAAGGAC-3′
*Wx1* (GRMZM2G024993)	Forward:	5′-GTAGTAGGGGCTGACGGTGAG-3′
	Reverse:	5′-CTGAACCTCCCGGAGAGATTC-3′

### Thiamin analysis

Young maize leaf tissue (0.5 g) and anthers, silks, glumes, rachises, and rachillae (0.2 g) were flash frozen and ground to a fine powder in liquid N_2_. Three replicates of each sample were extracted in 500 μl of 7.2% perchloric acid by sonicating for 30 min in a bath, held on ice for 15 min with periodic vortex mixing, and then cleared by centrifugation at (14000 *g*, 10 min). The supernatant was then analyzed for thiamin and its phosphates by oxidation to thiochrome derivatives followed by HPLC with fluorometric detection (Fraccascia et al., 2007; Goyer et al., 2013).

### D_2_O labeling and LC-MS analysis

Young maize leaves (0.5 g) were cut horizontally into 5-mm wide segments, floated on 6 ml of 30% (v/v) D_2_O (Cambridge Isotopes) in H_2_O in a petri dish (60 × 15 mm) and incubated in continuous light (150 μE m^−2^s^−1^) for 24 h at 22°C. Control samples were incubated in H_2_O. Samples were then frozen in liquid N_2_ and stored at −80°C. Frozen leaves were ground in liquid N_2_and extracted in 7.2% perchloric acid as above. Thiamin phosphates were hydrolyzed to thiamin using potato acid phosphatase (Sigma Aldrich), as follows. To 90 μl of leaf extract, 13.5 μl of 5 M KOH was added and mixed well; the resulting potassium perchlorate precipitate was removed by centrifugation. Sodium citrate buffer (100 mM, pH 4.8, 100 μl) and 1 unit of potato acid phosphatase were then added followed by incubation at 37°C for 1 h. After deproteinization in a Centricon NMWL 10000 unit, thiamin was converted to thiochrome and separated by HPLC as above. Thiochrome-containing HPLC fractions were collected and concentrated *in vacuo*. LC/MS analysis was performed on an LTQ Velos mass spectrometer with Accela 600 UHPLC (Thermo, San Jose, CA). Separation was achieved on a Thermo Hypersil Gold aQ column (50 × 2.1 mm, 1.9 μm) under gradient elution. Mobile phase A was 10 mM ammonium formate in water and mobile phase B was 0.1% formic acid in acetonitrile. The gradient began with 100% A for 1 min and decreased to 70% A over 8.5 min, holding for 0.5 min at 70% A then returning to initial conditions in 0.5 min and holding for 2.5 min to equilibrate. The mass spectrometer was operated in positive heated electrospray ionization with the following settings: 40 arb sheath gas, 10 arb auxillary gas, 3 kV spray voltage, 350°C spray temperature, 350°C heated transfer tube temperature and 2V of source collision induced dissociation. A full scan was collected from *m/z* 100–600 and a zoom scan was collected from *m/z* 260–270 to evaluate isotopic enrichment.

### Conflict of interest statement

The authors declare that the research was conducted in the absence of any commercial or financial relationships that could be construed as a potential conflict of interest.
