# Corrigendum: Divisions of labor in the thiamin biosynthetic pathway among organs of maize

**DOI:** 10.3389/fpls.2018.00148

**Published:** 2018-02-20

**Authors:** Jiahn-Chou Guan, Ghulam Hasnain, Timothy J. Garrett, Christine D. Chase, Jesse Gregory, Andrew D. Hanson, Donald R. McCarty

**Affiliations:** ^1^Genetics Institute and Horticultural Sciences Department, Institute of Food and Agricultural Sciences, University of Florida, Gainesville, FL, United States; ^2^Horticultural Sciences Department, Institute of Food and Agricultural Sciences, University of Florida, Gainesville, FL, United States; ^3^Department of Pathology, Immunology, and Laboratory Medicine, College of Medicine, University of Florida, Gainesville, FL, United States; ^4^Department of Food Science and Human Nutrition, Institute of Food and Agricultural Sciences, University of Florida, Gainesville, FL, United States

**Keywords:** thiamin biosynthesis, comparative transcriptomics, maize development, pollen development, meristem metabolism

In the original article, there was a mistake in Figure 6 as published. The scale of the y-axis was a factor of 10 too large. The corrected Figure 6 appears below. The authors apologize for this error and state that this does not change the scientific conclusions of the article in any way.

**Figure 6:**
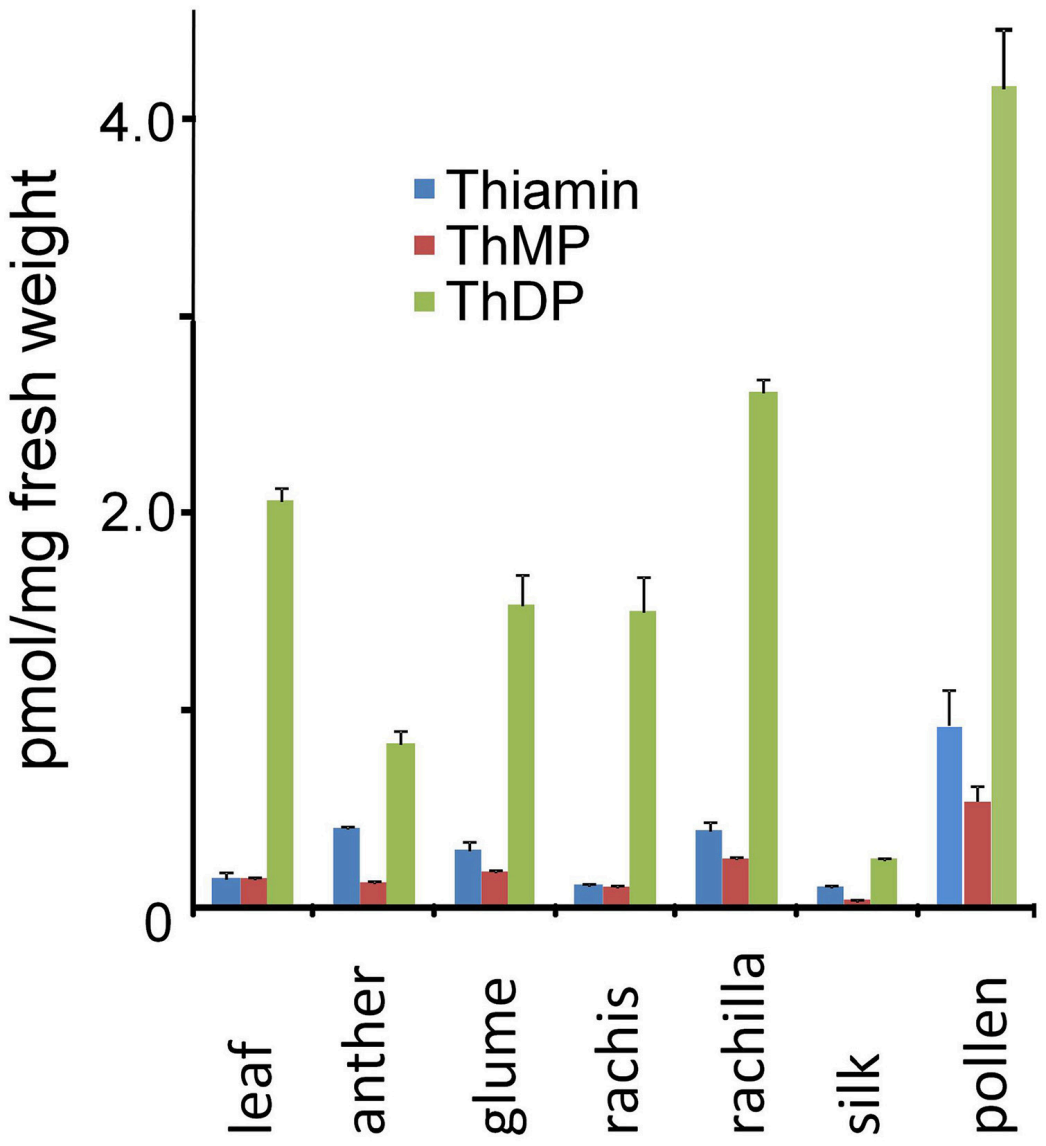
Thiamin and thiamin phosphate ester contents of maize leaves, inflorescence and floral organs. Thiamin content (pmol/mg of FW tissue) was determined as described in Methods. Bars: Standard error of the mean, *n* = 3.

The original article has been updated.

## Conflict of interest statement

The authors declare that the research was conducted in the absence of any commercial or financial relationships that could be construed as a potential conflict of interest.

